# Genetic Bone Diseases: A Scoping Review of Pathology, Symptoms, Diagnosis, Treatment, and New Horizons

**DOI:** 10.1002/ggn2.202500048

**Published:** 2026-06-23

**Authors:** Colin Jones, Ambalangodage C. Jayasuriya

**Affiliations:** ^1^ The Doctor of Medicine Program, College of Medicine and Life Sciences The University of Toledo Toledo Ohio USA; ^2^ Department of Orthopaedic Surgery, College of Medicine and Life Sciences The University of Toledo Toledo Ohio USA

**Keywords:** achondroplasia, craniosynostosis, genetic bone diseases, Marfan syndrome, osteogenesis imperfecta, osteopetrosis, Paget's disease

## Abstract

Genetic bone diseases are a rare group of afflictions suffered by the general population. However, their rarity should not diminish research efforts to help patients understand and treat their diseases. This review summarizes the pathology, symptoms, diagnosis, and treatment insight into six well‐known genetic bone diseases. Only six bone diseases are included due to the relatively low prevalence of them as whole limiting our scope to ensure accurate information and attention is provided for each disease individually. A literature search of PubMed is conducted, including studies published within the past five years (January 2020–December 2025). Thirty‐six studies met inclusion criteria, and no significant risk of bias is identified among the selected articles. Study findings are synthesized into disease overview, clinical and radiographic features, and diagnostic and treatment approaches. Actively developing or novel therapies relevant to each disease are also included. These treatments include: fresolimumab for osteogenesis imperfecta, small interfering ribonucleic acid (RNA) therapy for Osteopetrosis, denosumab for Paget's disease of bone, vosoritide/recifercept/infigratinib for achondroplasia, mesenchymal stem cell therapy for craniosynostosis, and combination losartan and atenolol therapy for Marfan syndrome. These treatments are generally more recently acknowledged in literature and are either actively undergoing research or require further research to determine their efficacy.

## Graphical abstract

This review highlights six genetic diseases of the bone, aiming to provide clinicians and researchers with updated information on their diagnosis and treatment. It also includes an assessment of common clinical and radiographic findings, along with pathophysiology related to diseases. The review specifically emphasizes novel treatments, including CRISPR/Cas9, as future avenues of research.

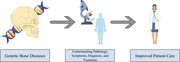

## 1 Introduction

**FIGURE 1:**
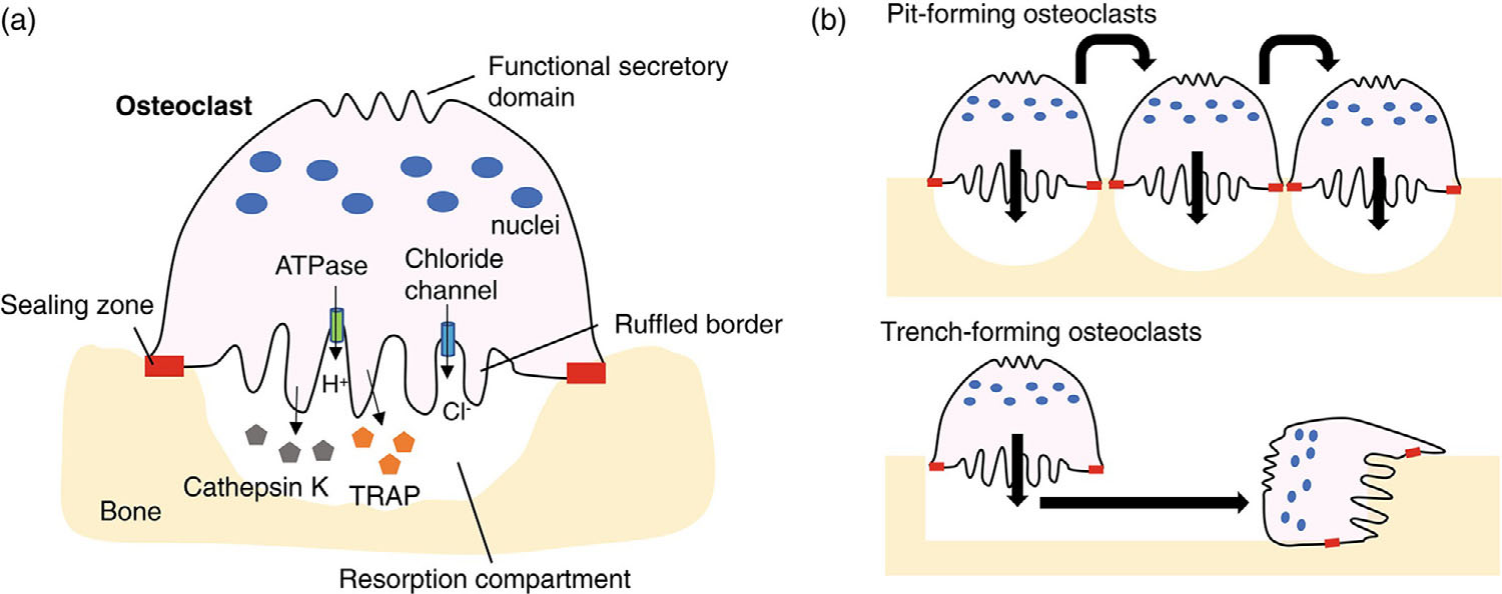
Osteoclast Function and Types [3]. Copyright Information: Creative Commons Attribution 4.0 License (CC BY) https://creativecommons.org/licenses/by/4.0/legalcode.en.

Genetic bone diseases are a rare but significant group of diseases comprising 5% of all birth defects [1]. Many genetic bone diseases, including osteogenesis imperfecta, osteopetrosis, and Paget's disease of bone, involve the improper activation of osteoclasts. Osteoclasts are a group of multinucleated bone cells that primarily break down the mineral and organic phases of bone [2]. They can form circular pits or longer trenches in the bone matrix, representing two different types of osteoclasts [3]. Pit‐forming osteoclasts resorb bone in a sporadic and stationary pattern, while trench‐forming osteoclasts resorb bone in a continuous pattern [3]. Cathepsin K and tartrate‐resistant acid phosphatase (TRAP) mediate matrix degradation, and these degradation products are endocytosed at the ruffled border to be later released at the functional secretory domain [3]. As osteoclasts maintain a balanced bone mass and density through resorption, osteoclast‐related pathology can be caused by under‐ and over‐activation. An overactivated osteoclast will resorb too much bone and cause a pathologically lower bone mass, while an under activated osteoclast will not resorb enough bone and lead to a pathologically increased bone mass. Figure 1 shows the osteoclast function and their types [3].

The remaining three pathologies in this review, craniosynostosis, achondroplasia, and Marfan syndrome, are connected to bone structure differently. Marfan syndrome is less explicitly connected to bone, instead involving a disease of connective tissue [4]. However, the skeleton is largely implicated along with the other non‐bone tissues. Craniosynostosis, a bone‐exclusive pathology, can have a variable cause: interleukin receptors, fibroblast growth factors, and transcription factors are among the many possible causes [5]. Achondroplasia involves chondrocyte dysfunction in the growth plates, leading to shortened extremities and other severe bone abnormalities [6].

This review seeks to highlight several of the comparatively well‐known genetic bone diseases. Specifically, this review will include achondroplasia, craniosynostosis, Marfan syndrome, osteogenesis imperfecta, osteopetrosis, and Paget's disease of bone. Given that the incidence of these diseases ranges from 1 in 66 in the case of Paget's disease of bone to 1 in 300 000 in the case of osteopetrosis (Table 1), a scoping review will provide useful information to clinicians seeking to understand disease characteristics that may not frequently occur in their patients. In terms of rare diseases, those that are known broadly have comparatively more literature available, leading to the selection of our chosen six for this review. However, because of the rarity in the general population, there is a still a concurrent lack of newly developing literature related to rare genetic bone diseases as a whole, as well as a lack of literature more broadly covering novel treatments. We are aiming to fill this gap in literature. Thus, our primary objective with this review is to provide the healthcare field with an easily accessible source of information for genetic bone diseases. Our secondary objective is to highlight several areas of novel treatment modalities that represent future research avenues that may be followed in the hopes of more effectively treating these diseases. These novel treatments include: fresolimumab for osteogenesis imperfecta, small interfering RNA therapy for osteopetrosis, denosumab for Paget's disease of bone, vosoritide/recifercept/infigratinib for achondroplasia, mesenchymal stem cell therapy for craniosynostosis, and combination losartan and atenolol therapy for Marfan syndrome. Further, we discuss the potential efficacy of CRISPR/Cas9 technology in treating these diseases, particularly osteogenesis imperfecta. No subgroup analyses were conducted between primary and secondary objectives due to the lack of data available in literature to compare how the accessibility of genetic bone disease information affects the usage frequency of novel treatments.

**TABLE 1:** Overview of genetic bone diseases.

Disease	Implicated gene(s) of interest (non‐exhaustive)	Common presentations (non‐exhaustive)	Incidence	References
Osteogenesis imperfecta	COL1A1, COL1A2	Multiple fractures with little/no trauma, blue sclera, bowing of long bones	1 in 10 000	[7]
Osteopetrosis	TCIRG1, OSTM1, PLEKHM1, SNX10, TNFSF11, TNFRSF11A, CLCN7, CA2	Multiple pelvic or spinal fractures, spine deformities, ‘erlenmeyer flask’ deformity of long bones	1 in 200 000 to 1 in 300 000	[8]
Paget's disease of bone	SQSTM1, TNFRSF11A/B, ZNF687, DCSTAMP	Bone pain, fractures, ‘blade of grass’ lesions in long bones, ‘Tam O’ Shanter Cap’	1 in 66 to 1 in 12	[9, 10]
Achondroplasia	FGFR3	Severely shortened stature, rhizomelic limb shortening, thoracolumbar kyphosis	1 in 25 000	[6, 11]
Craniosynostosis	TWIST1, IL11RA, FGFR1, FGFR2, FGFR3	Hydrocephalus, scaphocephaly, trigonocephaly, brachycephaly	1 in 2100 to 1 in 2500	[5]
Marfan syndrome	FBN1, TGFBR2	Tall stature, thoracolumbar scoliosis, pes planus, dolichocephaly	1 in 5000	[4]

## 2 Methods

### 2.1 Methodology

This review was completed by searching PubMed for relevant articles published in a peer‐reviewed journal, during the period of January 2020–December 2025. A single database, PubMed, was used given its large availability of research and ease of accessibility to physicians, researchers and laypeople alike. Further, PubMed will have a significant overlap of research with other public databases. Articles published more than 5 years prior to the writing of this review were excluded. This time frame was limited to 5 years to ensure that the most current data available was acquired for this review, as well as reducing the breadth of data that was retrieved and considered by the primary author. A small number of selected studies fall outside of the 2020–2025 timeline and were published in 2019. While these studies were originally within the 5‐year range of publication at the time of writing, the search window shifted during the editing process. Keywords searched included: “genetic” OR “heritable” AND “bone diseases”, “osteogenesis imperfecta”, “osteopetrosis”, “Paget's disease”, “craniosynostosis”, “achondroplasia”, and “Marfan syndrome”. Also searched were “osteoclasts”, “CRISPR/Cas9”, and “CRISPR/Cas9” AND “bone diseases”.

### 2.2 Eligibility Criteria, Study Selection, and Data Extraction

The articles published in the last five years were screened for their inclusion according to PRISMA guidelines. All retrieved articles were carefully reviewed by the primary author prior to applying the eligibility criteria. Study selection was based on predefined inclusion and exclusion criteria to promote transparency and consistency. Although the screening process was undertaken by a single reviewer and the review was not prospectively registered (e.g., PROSPERO), both authors jointly developed the eligibility criteria and reached consensus on the final set of included studies, thereby reducing the risk of selection bias. A total of 238,850 studies were identified using the above keywords in PubMed. The inclusion criteria for the selected studies were as follows: (i) genetic bone diseases, (ii) availability of clinical and radiographic presentations, (iii) information on diagnosis, and treatment of genetic bone diseases. The exclusion criteria were as follows: (i) eliminated studies published before 2020 and unavailable full texts using automation tools on PubMed, (ii) book chapters, and articles not relevant, (iii) reports not retrieved (*n* = 7), and (iv) due to redundant or unapplicable information (*n* = 10).

**FIGURE 2:**
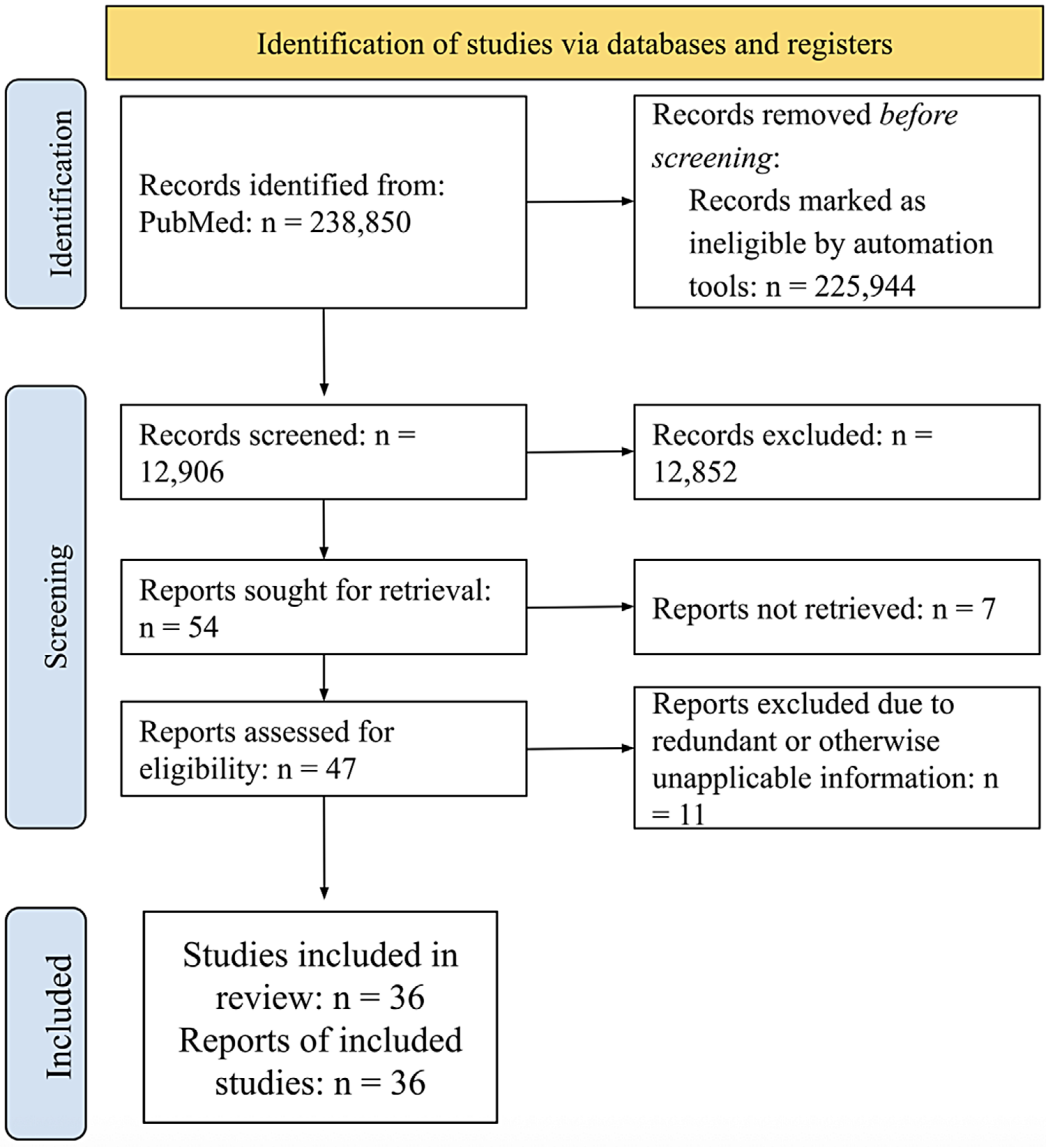
PRISMA‐Based Literature Selection Process. Copyright Information: Creative Commons Attribution 4.0 License (CC BY) https://creativecommons.org/licenses/by/4.0/.

Ultimately, 36 studies were selected. A schematic depicting the selection process is shown in Figure 2. Data extracted from each study correlated with the genetic basis, clinical and radiographic presentations, diagnosis, and treatment of each disease. Data was selected from each study based on its pertinence to the review objectives. Data was organized categorically as it related to each disease, and was further stratified into the aforementioned genetic basis, presentation, diagnosis, or treatment of each disease subsection. The synthesis was descriptive in nature, performed by the primary reviewer, with consistency maintained by oversight and assessment from the second reviewer. As the reviewed literature predominantly pertained to objective and well‐researched disease pathology, clinical manifestations, and diagnosis, bias was not determined to be a confounding factor in choosing literature for its inclusion in the review.

## 3 Results

### 3.1 Overview of Genetic Bone Diseases and Their Treatment

**FIGURE 3:**
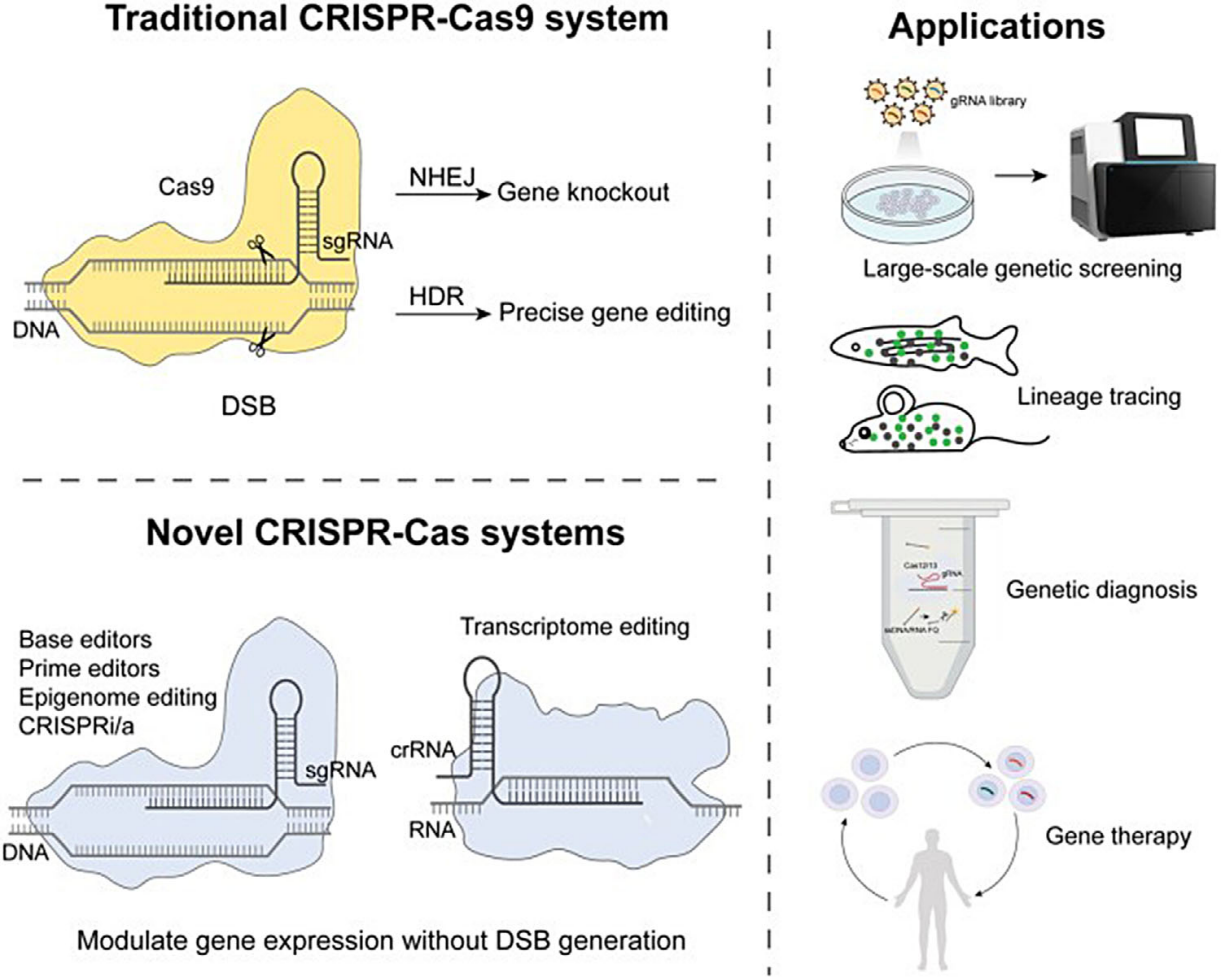
Use of CRISPR/Cas9 for Genetic Diseases [15]. Copyright Information: Creative Commons Attribution 4.0 License (CC‐BY) https://creativecommons.org/licenses/by/4.0/.

Table 1 lists the affected genes, common symptoms, and incidence of six genetic bone diseases covered in this review. Table 2 compiles novel treatments for each disease that may be pursued as future research directions. Table 3 highlights a comparison between the surgical and non‐surgical treatments. Future research should be undertaken to ensure the efficacy and continued success of known treatments, as recent data on this content is limited. Finally, Figure 3 highlights a schematic depicting usage of CRISPR/Cas9 gene editing as a potential diagnosis and treatment modality for genetic diseases, and by extension genetic bone diseases, which is discussed in depth in Section 4.2.

**TABLE 2:** Novel or developing treatments of genetic bone diseases.

Disease	Novel or developing treatment(s) of note	Relevant information	References
Osteogenesis imperfecta	Fresolimumab	Anti‐TGF‐β antibody originally used to treat cancer.	[12]
Osteopetrosis	Small interfering RNA therapy	Very little literature reported on the topic in the previous 5 years. Specific to ADO type II osteopetrosis.	[13]
Paget's disease of bone	Denosumab	Monoclonal antibody that suppresses osteoclasts and has been shown to improve symptoms.	[10]
Achondroplasia	Vosoritide, recifercept, infigratinib	MAPK pathway inhibitor, soluble FGFR3, and FGFR3 tyrosine kinase inhibitor respectively. Vosoritide approved for use in some countries, Recifercept and infigratinib undergoing phase II clinical trials.	[6]
Craniosynostosis	Mesenchymal stem cell therapy	Has been demonstrated to regenerate a functional suture and lessen negative neurological effects in a mouse model.	[5]
Marfan syndrome	Combination losartan and atenolol therapy	Separately shown to have similar efficacy in reducing aortic root dilation in Marfan Syndrome, but few studies conducted to determine the efficacy of combination therapy.	[14]

**TABLE 3:** Surgical and non‐surgical treatments.

Disease	Surgical treatments	Non‐Surgical treatments
Osteogenesis Imperfecta	Bone fracture and bowing repair	Bisphosphonates, romosozumab, CRISPR/Cas9
Osteopetrosis	Not Indicated	Hematopoietic stem cell transplants, SiRNA therapy, calcium, vitamin D
Paget's Disease of Bone	Correction of severe osteoarthritis	Bisphosphonates, calcitonin
Achondroplasia	Decompression of stenosed spine/foramen magnum, long bone lengthening	Vosoritide, recifercept, infigratinib
Craniosynostosis	Correction of head shape, endoscopic suturectomy	Remodeling helmet, mesenchymal stem cell therapy
Marfan Syndrome	Nuss procedure, substernal bar insertion, spinal stabilization	Leg bracing, combination losartan and atenolol

### 3.2 Osteogenesis Imperfecta

#### 3.2.1 Overview

Termed “brittle bone disease,” osteogenesis imperfecta (OI) is a genetic bone disease that manifests at an estimated rate of 1 in 10 000 [7]. OI received its colloquial name due to the high level of bone fragility and frequency of fractures associated with the disease [7]. OI presents in 21 different forms, with the most predominant phenotypes occurring due to the autosomal‐dominant inheritance of a deleterious COL1A1 or COL1A2 mutation [7]. COL1A1 and COL1A2 mutations affect the structure of type I collagen, particularly through the substitution of glycine with a different amino acid [7]. Thus, the type I collagen loses its appropriate function qualitatively. Approximately 10% of cases are associated with autosomal recessive, dominant, or X‐linked inheritance of non‐collagenous genes [7]. In the case of the predominant 90% of OI, pathogenicity is achieved through structural alterations of type I collagen, a key element in bone strength [7]. Carboxyl terminal glycine substitutions, specifically in COL1A1, are positively correlated with clinical severity [7]. Mild phenotypes of OI are associated with haploinsufficient mutations of only one gene [7].

#### 3.2.2 Clinical and Radiographic Manifestations

A distinguishing clinical manifestation of OI is bone fragility leading to high fracture incidence with little or no trauma [7]. In more severe forms of OI, progressive bowing of long bones, scoliosis and rib cage deformities are common [7]. Growth deficiency is present in moderate and progressively deforming OI but should be noted to also occur in mild type I OI [7]. Also prominent in type I OI is characteristic blue sclera and hearing loss onset in the second or third decade of life [7]. However, these symptoms can still be present in other types of OI. Other non‐bone manifestations include muscle weakness, cardiovascular difficulties, and pulmonary complications that increase in incidence with age [7].

Radiographic manifestations of OI predominantly fall into 3 categories: osteopenia/osteoporosis, bone deformities, and bone fractures [16]. Thinning of cortical bone and transparency of trabecular bone is typical of generalized osteopenia/osteoporosis. While these findings may be present in OI, it is important to differentiate low bone density as attributable to OI and not another cause [16]. Many bone deformities may be present on radiographic imaging, typically pertaining to the skull, long bones in the upper and lower limbs, chest, and spine [16]. The skull may radiographically present with supernumerary wormian bones that increase in frequency with the severity of OI [16]. Basilar impression may also be appreciated, along with rare but possible prominence of the occipital region, termed Tam O'Shanter Cap [16]. Slender long bones with bowing are typically present as well [16]. In the spine, kyphosis, scoliosis, codfish vertebrae and platyspondyly can be present [16].

#### 3.2.3 Diagnosis and Treatment

Diagnosing OI can begin as early as the first or second trimester of pregnancy [17]. If markers of OI are noted on prenatal ultrasound, magnetic resonance imaging (MRI), or computed tomography (CT), genetic testing can be utilized to diagnose OI [17]. Once this diagnosis is indicated, invasive (preferred) or noninvasive prenatal testing can confirm the diagnosis [17]. Postnatal diagnosis involves a consideration of family history and clinical and radiographic presentation [17]. Genetic testing is the only way to confirm OI as a diagnosis [12].

A primary mode of treatment for OI is to prevent bone fractures by increasing bone mass [18]. Bisphosphonates are typically used to this end by inhibiting osteoclast activity [18]. However, these drugs are only efficacious for a year of administration despite a long half‐life and are not efficacious in all patients [18]. Frequently administered to children, one clinical study looked at the effect of one such bisphosphonate, risedronate, on skeletal response to mechanical stimulation in children with OI [16]. Despite a small cohort of children, the study determined that risedronate significantly attenuates bone remodeling (formation and resorption) after the remodeling is activated by mechanical stimulation [16]. Other drugs are used to stimulate bone formation, including romosozumab, which inhibits a compound involved in signaling for osteoclast‐osteoblast stimulation [18]. Romosozumab shows potentially severe cardiovascular side effects among beneficial bone density increases [18]. While little contemporary research regarding romosozumab and OI is available, one case study found that the administration of 210 mg subcutaneous romosozumab significantly improved bone mineral density in an adult patient with OI type I and osteoporosis [19]. Side effects were not noted [19].

Fresolimumab represents a developing treatment with the potential to treat OI. Fresolimumab is a monoclonal antibody that neutralizes TGF‐ß heterodimers, which are implicated in pathogenesis of OI [20]. TGF‐ß maintains bone homeostasis by promoting osteoprogenitor differentiation and inhibiting osteoblasts [21]. In a recent phase I clinical trial, fresolimumab was shown to increase bone mass in a cohort of patients with moderate to severe OI [20]. While promising, the trial also noted that the dosing of fresolimumab should be correlated with the severity of TGF‐ß dysregulation and clinical severity [20].

### 3.3 Osteopetrosis

#### 3.3.1 Overview

Osteopetrosis presents in one of four inheritance patterns: autosomal dominant (ADO), Intermediate autosomal recessive (IAO), autosomal recessive (ARO), or X‐linked [8, 22]. ADO is the most common form of disease with an incidence rate of approximately 1 in 20 000 [22]. ARO is comparatively rare, with an incidence rate of 1 in 200,000 ‐ 300 000 [22]. IAO and X‐linked incidence is not well‐reported in recent literature. Pathogenesis of osteopetrosis, regardless of inheritance pattern, involves deficient osteoclast activity [8]. Deficient osteoclasts lead to increased bone density and brittleness, ultimately giving the disease its moniker “marble bone disease” [8]. Approximately 8 genes are implicated in most osteopetrosis pathology: TCIRG1, OSTM1, PLEKHM1, SNX10, TNFSF11, TNFRSF11A, CLCN7, and CAII [8]. Other undiscovered genes account for 30% of cases [8]. CLCN7, a gene encoding the chloride channel 7 protein, is implicated in both ARO and ADO [8]. Heterozygous mutations in CLCN7 lead to a loss of function and improper bone resorption by osteoclasts through the loss of a Cl‐/H+ antiporter [8]. Phenotype can vary massively even among patients with the same genotype. However, those with ARO and IAO tend to have the more severe phenotypes [8]. Mutations in TCIRG1, OSTM1, PLEKHM1, SNX10, TNFSF11, TNFRSF11A are seen in the malignant, fatal form of ARO, whereas loss of functions of CAII, a carbonic anhydrase, account for the onset of IAO symptoms in the first decade of life [8].

#### 3.3.2 Clinical and Radiographic Manifestations

Osteopetrosis is present clinically as highly variable depending on the inheritance pattern. The most common form, ADO, can present as entirely asymptomatic until adulthood, or can present with mild‐moderate symptoms, including multiple fractures of the spine and pelvic bones [8]. The rarer form, ARO, is much more severe and presents earlier [8]. Craniofacial morphology changes, blindness, facial palsy, and deafness are among the many symptoms common to infants and children [8]. The X‐linked form of osteopetrosis presents with the same symptoms in addition to immunodeficiency, lymphedema, and hair/skin/nail/sweat gland abnormalities [8].

Radiographically, osteopetrosis typically presents with any number of four classic features: diffuse increased bone density and sclerosis of the skull/spine/pelvis/appendicular bones, defects and alterations in long bone metaphysis, “bone‐in‐bone” appearance, and “sandwich‐vertebrae” or “rugger‐jersey” spine [8]. The increased bone density and sclerosis are found in all types of osteopetrosis [8]. Alterations in long bone metaphysis often present with a lack of concavity and widened appearance; this appearance is termed the Erlenmeyer flask deformity [8]. A “bone‐in‐bone” presentation is most frequently found in ADO type II [8]. “Sandwich‐vertebrae” occur due to well‐defined sclerosis and thickening of the vertebral endplate, whereas “rugger‐jersey” spine occurs due to ill‐defined sclerosis and increased density of vertebral endplates at multiple contiguous levels [8].

It should also be noted that the qualitative radiographic findings may in fact be correlated with quantitative clinical findings, particularly bone mass density. A 2022 study found that there was a significant positive correlation between radiographic and clinical severity of 12 patients with ADO type II [23]. In particular, quantitative CT of the lumbar spine strongly correlated with important clinical symptoms, including vision impairment and bone marrow failure [23].

#### 3.3.3 Diagnosis and Treatment

Osteopetrosis is diagnosed through a combination of radiological findings, clinical symptoms, and the presence of gene mutations [8]. A typical radiological finding can provide reason for a symptom or vice versa. Genetic testing may then confirm the type or inheritance pattern of osteopetrosis to guide further treatment and evaluate prognoses [8].

Treating osteoporosis involves monitoring disease severity and treating symptoms: calcium and vitamin D intake, neurological evaluations, ophthalmologic surveillance, and routine dental care may all be necessary [13]. For patients with severe ARO with bone marrow failure, hematopoietic stem cell transplants (HSCTs) are indicated [13]. Small interfering RNA (siRNA) therapy represents an unexplored avenue of treatment for ADO type II [13]. Given the difficulty of delivering siRNA to bone, recent literature has reported impressive efficacy of silicon stabilized hybrid lipid nanoparticles (sshLNPs) as a delivery method [24, 25]. sshLNPs have minimal liver accumulation and high efficiency of transfection, along with the ability to be topically or systemically administered [24]. In two separate mouse models, sshLNP delivery of siRNAs were shown to effectively knockdown mutant CLCN7 mice with ADO type II [24, 25]. Further, an improved understanding and classification of biomarkers associated with ADO type II would allow more efficient diagnosis and future mutation‐targeted siRNA therapy in humans [26]. Ultimately, the encouraging results from these studies reinforces the fact that further research into the applicability of siRNA therapy in humans is necessary.

### 3.4 Paget's Disease of Bone

#### 3.4.1 Overview

Paget's disease of bone (PDB) is primarily linked to genetics, with clinical variability likely due to environmental factors [9, 27]. While not all PDB cases can be accounted for due to genetic factors, this review will focus on the genetic basis for PDB, rather than the potential effect of the environment.

Initially termed “osteitis deformans” by Sir James Paget, PDB is the second most prominent disease of bone remodeling, with an incidence of approximately 1.5–8.3% worldwide [9]. PDB is primarily caused by improper osteoclast activation, erroneously increasing bone mass [9]. Structural abnormalities and weakened bone mass subsequently occur [9]. Up to one‐third of patients with PDB inherited the disease in an autosomal dominant pattern, and there is a higher incidence in men than women [9, 28]. Further, the prevalence of PDB increases with age starting at 50 years of age [28]. Heterozygous, rarely homozygous mutations in the SQSTM1 gene (encoding the p62 protein which plays a role in autophagy) are most often implicated in PDB cases: up to 50% of patients with familial PDB have a mutation in this gene [9]. Mutations in this gene, particularly a Pro392Leu mutation, lead to loss of function of osteoclast autophagy and accumulation of damaged proteins [9]. 13 other genes have been implicated in PDB pathogenesis, including but not limited to TNFRSF11A/B, ZNF687, and DCSTAMP [9]. The majority of these other genes are linked to osteoclast function or differentiation [9]. Phenotypic heterogeneity is possible even among patients with the same mutation [9]. Nonsense mutations causing partial translation of the UBA domain of SQSTM1 have the most severe phenotype [9]. Missense and somatic mutations in SQSTM1 comparatively have much less severe phenotypic presentations [9].

#### 3.4.2 Clinical and Radiographic Manifestations

In approximately 70% of patients, PDB is asymptomatic [9]. However, when symptoms are present, they primarily include bone pain, morphological conditions, hearing loss, and pathological fractures [9]. Bone deformities, including leontiasis ossea, can also occur [9]. Osteoarthritis may also present and lead to further pain in symptomatic patients [29]. The axial skeleton is often the affected area in PDB [9].

Radiographs of PDB patients can vary based on the progression of the disease [10]. In the osteoclastic phase of PDB, long bones present with V‐shaped “blade‐of‐grass” lesions, along with lucent zones of osteolysis in the skull [10]. As the disease progresses, bones demonstrate further osteolysis, thickening and coarsening of trabeculae occur, and vertebral bodies are enlarged throughout the spine [10]. Sclerosis is most prevalent as the disease reaches its most advanced phase [10]. The enlargement of the diploic space in the skull and spine may manifest and is referred to as a Tam O'Shanter Cap and “picture frame vertebra” respectively [10]. Further, long bone‐bowing deformities and accompanying stress fractures on the tension side of the bone may present [10].

#### 3.4.3 Diagnosis and Treatment

PDB is most simply diagnosed through a plain radiograph, with one of the many above manifestations present [10]. However, nuclear bone scan is the most sensitive test available to detect pagetic bone lesions [10]. Beyond this, CT or MRI scans may be helpful in patients with clinical findings but no radiographic findings [10]. Increased serum markers, particularly ALP, are frequently used to diagnose asymptomatic patients [10]. P1NP, serum CTX, urine NTX, and urine hydroxyproline will also be elevated in PDB patients.

Typically, only symptomatic patients necessitate treatment [10]. In the case of symptomatic patients or asymptomatic patients at risk of further PDB‐related complications, the treatment of choice is bisphosphonates [9, 10]. Bisphosphonates have been shown to normalize ALP levels and provide success for patients with moderate‐to‐severe PDB [9]. Zoledronic acid is often the therapy of choice when using bisphosphonates, despite potential renal toxicity [9]. A recent study found that the use of zoledronic acid (5 mg IV) showed an inexplicably higher elimination of pre‐existing bone lesions compared to the placebo group [30]. Calcitonin may be indicated in patients who cannot tolerate bisphosphonates [9]. Surgical intervention with preventative bisphosphonate therapy may also be utilized in cases of PDB‐related osteoarthritis [9].

Despite limited data, denosumab has been shown to normalize ALP levels in PDB patients following 4 to 8 months of recurrent subcutaneous injection [10]. Further improvements in symptoms and scintigraph findings were also reported, but a short pharmacological duration and frequent injects represent a possible barrier toward usage [10]. As of now, denosumab is not indicated for use in treatment of PDB [29].

### 3.5 Achondroplasia

#### 3.5.1 Overview

Achondroplasia is the most common form of skeletal dysplasia, occurring in 1/25 000 live births [11]. This condition is spurred by a heterozygous pathogenic gain‐of‐function mutation in the fibroblast growth factor receptor 3 (FGFR3) [11]. Through this gain of function, the MAPK signaling pathway is activated, which inhibits the proliferation of chondrocytes in the growth plate [6]. The most obvious effect of this mutation is severely short stature, 6–7 standard deviations below average height, along with a myriad of debilitating skeletal symptoms and complications [6, 11]. Achondroplasia is inherited in an autosomal dominant fashion, but 80% of cases are due to a de‐novo mutation associated with parental age > 35 years [6]. In AD achondroplasia, the vast majority (99%) of cases involve the same pathogenic variant of FGFR3 [6]. Among this pathogenic genotype, phenotypic presentation does not vary [6]. Those who inherit two copies of the mutated FGFR3 are unviable [6].

#### 3.5.2 Clinical and Radiographic Manifestations

Common clinical findings in achondroplasia include rhizomelia, macrocephaly, frontal bossing, and a depressed nasal bridge [11]. These findings are often present at birth or in early childhood, and due to the plainness of these clinical findings, a radiographic survey is usually not required [6, 11]. However, when radiographic studies are completed, findings may include square pelvis shape with horizontal acetabula, short vertebral pedicles with lower thoracic/lumbar interpedicular narrowing, proximal shortening of long bones, proximal femoral radiolucency, chevron shape of the distal femoral epiphyses, and thoracolumbar kyphosis [6]. As achondroplasia patients continue to age, they may be subject to serious orthopedic complications, including foramen magnum stenosis, deformities of the spine, and genu varum [11].

#### 3.5.3 Diagnosis and Treatment

Diagnosing achondroplasia can begin as early as the 12th week of pregnancy using chorionic villus sampling for genetic testing assuming that achondroplasia is strongly suspected due to genetic predisposition (i.e., the parents have achondroplasia) [6]. Without a positive family history, however, achondroplasia is much more difficult to diagnose until the 26th week of gestation [6]. At this point in the third trimester, a diagnosis can be made using sonography [6].

Postnatally, achondroplasia primarily involves a clinical and radiographic diagnosis based on findings described in the previous section. However, further genetic testing can be completed to corroborate clinical findings [6]. Atypical presentations of achondroplasia can involve next‐generation sequencing techniques [6].

Treatment of achondroplasia is largely based on symptom presentation [11]. Surgical intervention is commonly involved decompressing the spine or foramen magnum that has undergone stenosis. Additionally, surgical lengthening of long bones may be used to improve functionality and quality of life but is associated with complications [6, 11]

There is a severe lack of medicinal treatment options available for achondroplasia patients [11]. While growth hormone has historically been utilized as a treatment in Japan, it has debatable efficacy [11]. There are, however, several drugs being researched that may prove to aid in achondroplasia comorbidities [6]. Vosoritide, a C‐type natriuretic peptide and inhibitor of the MAPK pathway at the level of RAF‐1, has been authorized for use in children with achondroplasia in the European Union, United States, and Brazil as of 2021 [6, 11]. Compared to a placebo, vosoritide has been demonstrated to increase growth velocity and improve body proportions at significant levels [6]. Recifercept, which is soluble FGFR3, and infigratinib, an FGFR3 Tyrosine Kinase inhibitor, are both undergoing phase II trials to demonstrate their efficacy in treating achondroplasia [6].

### 3.6 Craniosynostosis

#### 3.6.1 Overview

Craniosynostosis is a congenital and pathogenic premature fusion of cranial sutures with an incidence between 1/2100 and 1/2500 in the United States [5]. Cranial sutures connect skull bones and allow the development of the skull and brain [5]. These sutures fuse at varying times throughout a person's life, ranging from 9 months to approximately 60 years [5]. In craniosynostosis, the sagittal, coronal, metopic, and lambdoid sutures are implicated [5]. In particular, the sagittal suture is implicated most frequently in 45% of cases in the United States, followed by coronal, metopic, and lambdoid sutures in order of descending frequency [5]. Craniosynostosis can present in multiple types and associated diseases: autosomal dominant inheritance of these diseases is the most common, followed by autosomal recessive and cross‐linked dominant inheritance patterns [5]. Clinical symptoms will vary depending on the type of craniosynostosis inherited [5].

Given the large number of craniosynostosis types, several mutations may contribute to the development of craniosynostosis [5]. Of note, heterozygous loss‐of‐function mutations in TWIST1, a gene encoding a loop‐helix‐loop transcription factor, will result in Saethre‐Chotzen syndrome due to a loss of osteogenic‐non‐osteogenic boundary and ossification of coronal structure [5]. Missense mutations in interleukin‐11 receptor alpha subunit, IL11RA, are attributable to Crouzon‐like craniosynostosis with multiple suture fusions [5]. Gain‐of‐function mutations in fibroblast growth factor receptors 1, 2, and 3 (FGFR1/2/3) are implicated in several craniosynostosis syndromes due to FGFR's role in skeletal homeostasis [5]. Gain‐of‐function mutations in RUNX2, a regulator of osteoblast differentiation, has also been shown to be implicated in craniosynostosis [5]. Ultimately, craniosynostosis has a high phenotypic variability, with each phenotype depending heavily on the genotype [5]. Compared to other genetic bone diseases, there is much less phenotypic variation among each individual genotype.

#### 3.6.2 Clinical and Radiographic Manifestations

Due to the sheer volume of syndromes associated with craniosynostosis, clinical manifestations may vary considerably. In sagittal suture fusions, scaphocephaly is the primary presentation [5]. Unilateral coronal suture fusion is associated with ipsilateral forehead flattening and rotation of the midface [5]. Bilateral coronal suture fusion is associated with brachycephaly [5]. Metopic suture fusion presents with trigonocephaly [5]. Unilateral lambdoid suture fusion results in mastoid process bulging, cranial base lowering, and the inferior/posterior displacement of the ear [5]. Lastly, bilateral lambdoid suture fusion presents with a flat, widened occipital region and inferior/anterior ear displacement [5]. Symptoms that are not unique to any one type of suture fusion include hydrocephalus, hearing and visual problems, heightened intracranial pressure, and intellectual disabilities [5].

These hallmark manifestations can also be found radiographically as they are found clinically. Further, imaging modalities can be used to identify suture patency and structural abnormalities in the brain [31]. CT scans remain the gold standard for imaging craniosynostosis, but ultrasound and MRI imaging are gaining traction as non‐ionizing alternatives [32].

#### 3.6.3 Diagnosis and Treatment

Diagnosis of craniosynostosis is primarily clinical and frequently occurs before the age of one [31]. Clinicians may include considerations of family history of unusual head shapes, teratogen exposure, pregnancy complications, and evidence of achieved childhood milestones [31]. For craniosynostosis associated with other syndromes, congenital abnormalities are also considered [31]. Sutural ridging, scalp blood vessel prominence, and size/shape of the fontanels should also be considered [31]. Radiographic imaging may then be used to confirm any clinical diagnosis [31].

Surgical intervention is the most common treatment for craniosynostosis, particularly that of the syndromic type [31]. In the case of normal intracranial pressure, the optimal time for surgery is between the ages of 6 and 12 months, with the ultimate goal of achieving normal brain development and a more cosmetically appreciable appearance [31]. A postoperative remodeling helmet may be indicated for younger children [31]. Children with non‐syndromic or uncomplicated cases of craniosynostosis may choose not to do treatment or take a more conservative treatment approach, involving a remodeling helmet or endoscopic suturectomy [31].

Many prospective non‐surgical therapies represent novel ways of treating craniosynostosis. Mesenchymal stem cells can be an effective treatment based on a mouse model of Saethre‐Chotzen syndromic craniosynostosis [5]. Recombinant periostin has been shown to mitigate coronal craniosynostosis in the same mouse model, and GSK‐J4, an inhibitor of Kdm6a and Kdm6b, also prevented coronal suture fusion in this model [5].

### 3.7 Marfan Syndrome

#### 3.7.1 Overview

Marfan syndrome (MFS) is a connective tissue disease with an incidence of 1 in 5000 [4]. It is inherited in an autosomal dominant fashion, but can also be caused by a sporadic mutation [4]. Classical MFS accounts for 90% of cases and involves missense or nonsense mutations in FBN1 which encodes fibrillin‐1 [4]. MFS type 2, characterized by a comparatively more severe cardiovascular involvement, is caused by mutations in the gene for transforming growth factor beta receptor II (TGFBR2) [4]. MFS most commonly involves FBN1 point mutations, often causing the substitution of critical cysteine residues [4]. This leads to a loss of function in fibrillin‐1 and the characteristic symptoms of MFS [4]. FBN1 mutations do not appear to have strict correlation between genotype and phenotype, but the strongest correlation lies in mutations in FBN1 exons 24–32 [4]. Other phenotypic variations can be attributed to environmental factors or modifying genes [4]. Early diagnosis and treatment lead to similar prognoses for patients with both classical and type 2 MFS, but the often‐later diagnosis of MFS type 2 typically leads to a worse prognosis.

In classical MFS, mutations in fibrillin‐1 leaves it proteolytically susceptible, eventually leading to aortic stiffness and reduced elasticity of the aortic wall [4]. Further, there will be a phenotypic change of smooth muscle cells to a proliferative state, causing a loss of smooth muscle cell contractility and aorta dilation [4]. The altered smooth muscle cells also induce the loss of elastin fibers, causing aortic aneurysms and valve lesions [4]. Fibrillin‐1 also plays a role in limiting the activity of TGF‐beta [4]. When not properly regulated, as in the case of MFS, overactivation of TGF‐beta induces many of the characteristic phenotypic presentations of MFS: developmental emphysema, mitral valve prolapses, and aortic aneurysm formation/myopathy [4].

#### 3.7.2 Clinical and Radiographic Manifestations

While the pathogenesis of MFS correlates with cardiovascular problems, fibrillin‐1 and microfibrils play a critical role in bone formation and function [33]. For this review, we will focus on the skeletal manifestations of MFS.

The main skeletal presentation of MFS is the excessive growth of limb bones, leading to tall stature and an arm‐span to height ratio > 1.05 [4]. A majority of MFS patients experience thoracolumbar scoliosis [4]. Pes planus, protrusio acetabuli, and camptodactyly are also common [4]. Several facial characteristics are frequently present, including dolichocephaly, high arched palate, dental crowding, retro‐ or micrognathia, malar flattening, and palpebral fissures [4]. Many patients also develop degenerative arthritis earlier than expected [33]. However, it should be noted that the common skeletal abnormalities seen in MFS are true for adults of European descent but have not been accurately confirmed for all ethnicities [33]. In particular, Hispanic and Asian adults often lack significant skeletal abnormalities despite presenting with other facets of MFS [33]. However, these patients continue to exhibit ocular and aortic complications at the same rate as MFS patients of European descent [33]. Thus, Asian and Hispanic patients may be less likely to be diagnosed with MFS based on the clearly observable skeletal findings [33].

Repeated imaging in MFS primarily pertains to the aorta to prevent dissection [33]. CT angiography, transthoracic echocardiography, and MRI imaging modalities are used for this purpose [33]. In the case of skeletal abnormalities, radiographic imaging can confirm a clinical diagnosis but will not necessarily show any different manifestations than those described above.

#### 3.7.3 Diagnosis and Treatment

The most recent diagnostic criteria for MFS were introduced in 2010 and are termed Ghent II nosology [33]. The Ghent II criteria include the following manifestations, with each assigned a varying amount of points: wrist and thumb signs (3), wrist or thumb sign (1), anterior chest deformity (2), hind foot deformity (2), pneumothorax (2), dural ectasia (2), protrusio acetabuli (2), reduced upper segment/lower segment and increased arm span:height ratio (1), reduced elbow extension (1), non‐pregnancy/obesity induced skin striae (1), myopia more than 3 diopters (1), and mitral valve prolapse (1) [33]. Also, worth a single point is if 3 out of 5 of the following facial features are present: dolichocephaly, enophthalmos, down slanting palpebral fissures, malar hypoplasia, and retrognathia [33]. Consequently, if 7 or more points are acquired and there is a family history of MFS or an aortic root dilation, a diagnosis of MFS can be made [33]. Ectopia lentis or a FBN1 can also be used in place of the aforementioned criteria to confirm an MFS diagnosis [33]. Further, genetic testing should be utilized to rule out a similarly presenting syndrome [33].

Skeletal complications of MFS are treated as they arise [33]. A minimally invasive surgical approach, the Nuss procedure, is utilized to repair deformities to the anterior chest and spine with success in most patients [33]. However, severe deformity requires inserting a substernal bar [33]. As MFS can lead to severe spinal deformity and associated discrepancy in leg length, external bracing may be utilized in children with progressing spinal deformities [33]. If scoliosis increases beyond 40 degrees, surgical stabilization of the spine should be considered [33].

ß‐blockers, namely atenolol, have been considered the standard therapy for MFS patients with aortic root dilation [14]. Based on this information, another class of antihypertensive medication, angiotensin‐II receptor blockers (ARBs), was examined for uses in MFS. Specifically, losartan was shown in animal studies to aid in combatting MFS symptoms [14]. In more recent studies, losartan was shown to not significantly differ in efficacy when compared to atenolol [14]. Thus, as the two medications can be safely utilized simultaneously, combination atenolol and losartan therapy represents an unexplored treatment avenue for MFS patients [14]. So far, two small studies have shown benefit in utilizing this drug combination, and any drug therapy that involves losartan may be more effective in treating MFS patients that are FBN1 haploinsufficient [14].

## 4 Discussion

### 4.1 Overview of Findings

#### 4.1.1 Comparison with Existing Literatures

The main implication of our review is that more research must continually be done on these diseases, regardless of their perceived or actual rarity in a clinical setting. As a whole, the pathophysiology, symptoms, and diagnosis that we have reported aligns with the historical clinical management of our selected diseases. However, where our findings diverge from historical data lies in the treatment modalities, including CRISPR/Cas9. The emphasized novel treatments are reported in the literature, but not extensively. Thus, our review provides an up‐to‐date source of treatments for clinicians seeking the most current options for their patients.

#### 4.1.2 Clinical Implications

In terms of treatment, we would like to emphasize that clinicians should not abandon gold standards. The main recommendation from this review is to consider novel treatments in symptomatic patients that are refractory to standard treatment modalities, or as future research topics to eventually improve the quality of life for genetic bone disease patients.

#### 4.1.3 Limitations

In the past 5 years, little research has been conducted on many of the diseases highlighted in this review. Thus, some resources had to be utilized multiple times to convey information about a single genetic disease. Further, this review was limited to a single database, which may have curtailed the available information to be reported. As study screening was conducted by only one reviewer, both selection and interpretation bias are potentially present. The bias was mitigated through thoughtful assessment by a second reviewer. However, these limitations are impossible to fully mitigate. Thus, the overall confidence in findings may be slightly less than complete. Readers should interpret these conclusions using their own due diligence to ensure accuracy of the information. We would also like to reiterate the nature of this scoping review: it is intended to inform, rather than provide quantitative conclusions in regards to the diseases or treatments.

### 4.2 Genetic Editing as Novel Treatment and Call to Action

As each of these diseases occur due to genetic mutation, genome editing represents a novel potential for treatment that to prevent or more effectively treat these conditions. The clustered regularly interspaced short palindromic repeats (CRISPR)/Cas9 system could be utilized to this extent due to its high relative efficiency and accuracy [34]. A schematic depicting the process in which CRISPR/Cas9 could be utilized in genetic disease treatment is shown in Figure 3 [15]. CRISPR/Cas9 is a genome editing technology that involves the use of a single guide RNA and CRISPR‐associated protein 9 (Cas9) nuclease [15]. For bone repair, CRISPR/Cas9 can be utilized to produce patient‐derived pluripotent stem cells to differentiate into bone or cartilage [35]. As such, the CRISPR/Cas9 system may be incredibly valuable in genetic bone diseases that are monogenic, like OI [35]. A 2021 study utilized CRISPR/Cas9 to correct a COL1A1 gene mutation in induced pluripotent stem cells (iPSCs) of OI patients [35]. This process recovered collagen I expression and rectified the osteogenic potential of the iPSCs [35]. Further, CRISPR/Cas9 has been used in a mouse model to effectively treat and improve bone architecture and spontaneous fractures in a COL1A2 mutant OIM that resembles severe dominant type III OI in humans [36]. Using recombinant adeno‐associated virus, CRISPR/Cas9 was delivered to skeletal osteoblast‐lineage cells [36]. This reversed osteogenic differentiation dysregulation caused by the COL1A2 mutation, lowered bone turnover rates, improved grip strength, and improved skeletal deformities [36].

While the recent research into the topic of CRISPR/Cas9‐based OI treatment seems promising, the fact remains that very little research has been conducted on this topic. The extent to which CRISPR/Cas9 can benefit humans with different types of OI remains to be seen, let alone using CRISPR/Cas9 gene editing as a standard therapy for OI. Further, the application of CRISPR/Cas9 gene editing has not been sought regarding other genetic bone diseases. It could be utilized by reversing the FBN1 mutations in MFS patients, or perhaps in the SQSTM1 gene often mutated in PDB patients. The boundless utilization possible for the CRISPR/Cas9 system lies beyond the scope of this review. However, we provide a call to action to continue contemporary research and bolster future research efforts toward finding the positive implications of the CRISPR/Cas9 system in genetic bone diseases

## 5 Conclusion

Genetic bone diseases are a group of undoubtedly rare diseases. They typically involve significant skeletal structure abnormalities and may drastically alter the patient's quality of life. Despite the low statistical likelihood of having a genetic bone disease, a sizable cohort of people will experience the negative effects of these pathologies. However, there is continued effort to aid these patients. New treatment methods, like the usage of siRNA therapy for osteopetrosis or the exciting proposition of CRISPR/Cas9 editing for OI and beyond, give hope to those who suffer from these genetic ailments. Clinicians and researchers must take it upon themselves to continue seek knowledge about the many genetic bone pathologies, including the six discussed in this review. The continuance of this research is critical to aid patients managing a severe illness caused by genetic factors beyond their control.

## Author Contributions

Colin Jones performed the data analysis, writing, editing, and reviewing the manuscript and Ambalangodage C. Jayasuriya performed writing, editing, and reviewing the manuscript.

## Conflicts of Interest

The authors declare no conflicts of interest.

## Data Availability

Data sharing is not applicable to this article as no new data were created or analyzed in this study.
